# Pathological characteristics of reoperated regrowing clinically nonfunctioning pituitary tumor cases in comparison with initial surgical cases

**DOI:** 10.3389/fendo.2024.1400671

**Published:** 2024-05-28

**Authors:** Atsushi Ishida, Naoko Inoshita, Noriaki Tanabe, Koji Takano, Sachiko Tanaka-Mizuno, Masataka Kato, Haruko Yoshimoto, Hideki Shiramizu, Go Matsuoka, Shozo Yamada

**Affiliations:** ^1^ Hypothalamic and Pituitary Center, Moriyama Memorial Hospital, Tokyo, Japan; ^2^ Department of Pathology, Moriyama Memorial Hospital, Tokyo, Japan; ^3^ Department of Endocrinology, Moriyama Memorial Hospital, Tokyo, Japan; ^4^ Laboratory of Epidemiology and Prevention, Kobe Pharmaceutical University, Kobe, Japan

**Keywords:** nonfunctioning pituitary tumor, regrowth, gross total resection, transcription factor, silent gonadotroph tumors, silent corticotroph tumor, null cell tumor

## Abstract

**Objective:**

Postoperative nonfunctioning pituitary tumor (NFPT) regrowth is a significant concern, but its predictive factors are not well established. This study aimed to elucidate the pathological characteristics of NFPTs indicated for reoperation for tumor regrowth.

**Methods:**

Pathological, radiological, and clinical data were collected from patients who underwent repeat operation for NFPT at Moriyama Memorial Hospital (MMH) between April 2018 and September 2023. For comparison, we also gathered data from patients who underwent initial surgery for NFPT during the same period at MMH.

**Results:**

Overall, 61 and 244 NFPT patients who respectively underwent reoperation and initial operation were evaluated. The mean period between the previous operation and reoperation was 113 months. Immunonegativity for any adenohypophyseal hormone was significantly more frequent in the reoperation group than in the initial operation group. In addition, the rate of hormone-negative but transcription factor–positive (H-/TF+) tumors among silent gonadotroph tumors was significantly higher in the reoperation group than in the initial operation group. Furthermore, seven silent corticotroph tumors (SCTs) in the reoperation group were ACTH-negative but TPIT-positive. Because most of the previous surgeries were performed in other hospitals a long time ago, we could procure the previous pathological results with immunohistochemistry (IHC) only from 21 patients. IHC for TF had not been performed in all the previous specimens. IHC for adenohypophyseal hormone was almost the same as the current results, and many H-/TF+ tumors were previously diagnosed as NCT. In addition, the reoperated patients were classified into 3 groups on the basis of the condition of the previous operation: gross total resection (GTR), 12 patients; subtotal resection (STR), 17 patients; and partial resection (PR), 32 patients. The mean Ki-67 LI in the GTR, STR, and PR subgroups were 1.82, 1.37, and 0.84, respectively, with the value being significantly higher in the GTR subgroup than in the PR subgroup (P < 0.05).

**Conclusions:**

The ratio of H-/TF+ tumors is significantly higher in symptomatically regrown tumors than in the initial cases, which used to be diagnosed as NCT. PR cases tend to grow symptomatically in a shorter period, even with lower Ki-67 LI than GTR cases.

## Introduction

Nonfunctioning pituitary tumors (NFPTs) are mostly benign and lack the clinical symptoms of excessive pituitary hormone levels. NFPTs usually show symptoms owing to the mass effect of the tumor, such as hypopituitarism and/or visual disturbance (1–3). Transsphenoidal surgery (TSS) is the gold standard for NFPT treatment (4, 5). After TSS for NFPT, recurrence is defined as the re-emergence of tumors from the absence of residual tumors on postoperative magnetic resonance imaging (MRI), whereas regrowth indicates that the remaining tumors after operation show enlargement during follow-up. The complete resection of NFPT at the cellular level is difficult, and theoretically, some remaining tumors usually exist and may regrow. Therefore, we used the term “regrowth” for “recurrence or regrowth” in this report. The symptomatic regrowth of NFPTs is not uncommon, but management is generally challenging because reoperation is more complicated and technically difficult than the initial operation (6). Furthermore, there is no effective medical therapy for most NFPTs. Thus, the characteristics of tumors that tend to regrow after operation are of great interest to pituitary surgeons. However, no histological predictive factors for NFPT regrowth have been identified.

The 4th edition of the World Health Organization (WHO) classification was updated to include immunohistochemistry (IHC) for transcription factors (TFs), such as steroidogenic factor 1 (SF1), T-box transcription factor (TPIT), and pituitary transcription factor 1 (PIT1), to classify NFPT [7]. The null cell tumor (NCT) was defined as immune-negative NFPT (i.e., negative transcription factors) in the 5th edition of the WHO classification (7–9). Since then, several studies have reported a relationship between these histological characteristics and regrowth rates (10–16). However, evidence is still controversial owing to the technical difficulty of IHC and the lack of a sufficient number of reoperated NFPT cases (10–16). Previous studies emphasized that gross total resection (GTR) in the initial operation was essential to prevent regrowth (17, 18). Thus, the better the surgical skills of the surgeon, the fewer regrowth occur. However, there are no reports of many reoperated cases, especially those with the abovementioned pathological characteristics. In addition, NFPTs with Ki-67 labeling index (LI) > 3% are at high risk of regrowth (19, 20). However, the Ki-67 LI of most NFPTs is <3% (21, 22); thus, the majority of regrowing NFPTs should have a low Ki-67 LI.

We encountered many cases of NFPT that regrew and became symptomatic after previous surgery. The senior author (S.Y.) reoperated on these cases, and an endocrine pathologist (N.I.) examined the extracted specimens and those from the initial surgical cases. Thus, we took advantage of this situation and studied the characteristics of reoperated NFPT specimens in a large population, and compared them with those of initial surgical cases.

## Methods

### Study design and population

This retrospective study was approved by the ethical review board of Moriyama Memorial Hospital (MMH) (Approval No. 23013) and was conducted according to the tenets of the Declaration of Helsinki. All patients with NFPT who underwent TSS for MMH between April 2018 and September 2023 were evaluated. Data, including radiographic, operative, and pathological findings, were obtained from medical records. Patients with information from previous hospitals or sufficient pathological examinations at MMH were included.

### Patient classification

The patients were divided into two groups: the reoperation group and the initial operation group. The reoperation patients underwent one or more surgeries, mostly in other hospitals. These patients were referred to our senior author for repeat operation to ameliorate the relapsed symptoms years after the previous operation. The reoperation group was further classified into three subgroups on the basis of the extent of resection (EOR) in the previous operation assessed by postoperative MRI and surgical record. Those with no detectable residual tumor on MRI were categorized into the GTR subgroup. Most of the tumors were resected in the subtotal resection (STR) subgroup, but a residue of <10% was present on postsurgical MRI. For the partial resection (PR) subgroup, ≥10% of the tumor remained after the operation (23).

### Pathological examination

The tissue specimens were evaluated using routine pathological and immunohistochemical examinations. Sections were incubated with the following antibodies for immunohistochemical evaluation: cytokeratin (CAM 5.2, Roche Diagnostic, Basel, Switzerland), growth hormone (MU925–5UC, BioGenex, Fremont, CA, USA), Ki-67 (MIB-1, Dako, Carpinteria, CA, USA), prolactin (PRL/2644, BioGenex, Fremont, CA, USA), adrenocorticotrophic hormone (ACTH) (02A3, Dako, Carpinteria, CA, USA), luteinizing hormone (C93, Dako, Carpinteria, CA, USA), follicle-stimulating hormone (C10, Dako, Carpinteria, CA, USA), thyrotropin stimulating hormone (0042, Dako, Carpinteria, CA, USA), PIT1 (D-7, Santa Cruz Biotechnology, Dallas, TX, USA), TPIT (CL6251; Atlas Antibodies, Bromma, Sweden), and SF1 (N1665; Perseus Proteomics, Tokyo, Japan). IHC studies were performed using a BenchMark GX automated slide preparation system (Ventana, Roche, Basel, Switzerland) according to the manufacturer’s protocols, except for SF1, in which samples were incubated overnight. Staining results were considered positive when >1% of the cells were positive for adenohypophyseal hormones; other patients were classified as hormone-negative tumors (HNTs) and examined also for TFs. Among them, tumors with immunoreactivity for TFs were named as hormone-negative but TF–positive (H-/TF+) tumors. In addition, the Ki-67 LI was measured in 1000 tumor cells, and the positivity rate was noted.

### Statistical analysis

Clinicodemographic patient factors were described using summary statistics. Categorical variables were presented as numbers and proportions, whereas continuous variables were presented as means and ranges. The immunohistochemical results were compared between the reoperation and initial operation groups by using Pearson’s chi-square test. The mean of Ki-67 LI and time until reoperation were compared among the three subgroups. Comparisons were made for each of the two subgroups by using Tukey’s test, with consideration to the multiplicity of statistical tests. All statistical analyses were performed using SAS version 9.4 (SAS Institute Inc., Cary, NC, USA). P-value < 0.05 was considered significant.

## Results

### Patient characteristics

The reoperation group involved 61 patients; 12, 17, and 32 patients were classified into the GTR, STR, and PR subgroups, respectively, based on the EOR in the previous operation. A total of 13/61 patients had undergone previous surgeries performed by the senior author (11 GTR patients and 2 STR patients). Previous surgeries of the remaining 48 patients were performed in other hospitals, and they were referred to us for repeat surgery to ameliorate imminent symptoms. We could procure the previous pathological results with reliable IHC from 21 patients among the reoperated 61 patients. IHC for TF was not studied in those patients because the initial surgeries were performed a long time ago. Meanwhile, the initial operation group performed in our hospital consisted of 244 patients. All surgeries of both the reoperation and the initial operation groups were performed by the same senior author. Demographics of patients in reoperation and initial operation groups were comparable and summarized in **Table 1**.

**Table 1:** Patient demographics of reoperated cases and initial cases.

	Reoperated cases (n=61)	Initial cases (n=244)	p-value
Mean age (y) (SD)	58.2 (12.7)	54.9 (14.0)	0.093
Gender, % of female	45.9	55.0	0.251
Mean value of Ki-67 LI (SD)	1.19 (1.0)	1.37 (2.2)	0.378

SD, standard deviation; Ki-67 LI, Ki-67 labeling index.

### IHC for adenohypophyseal hormones and TFs

**Figure 1:**
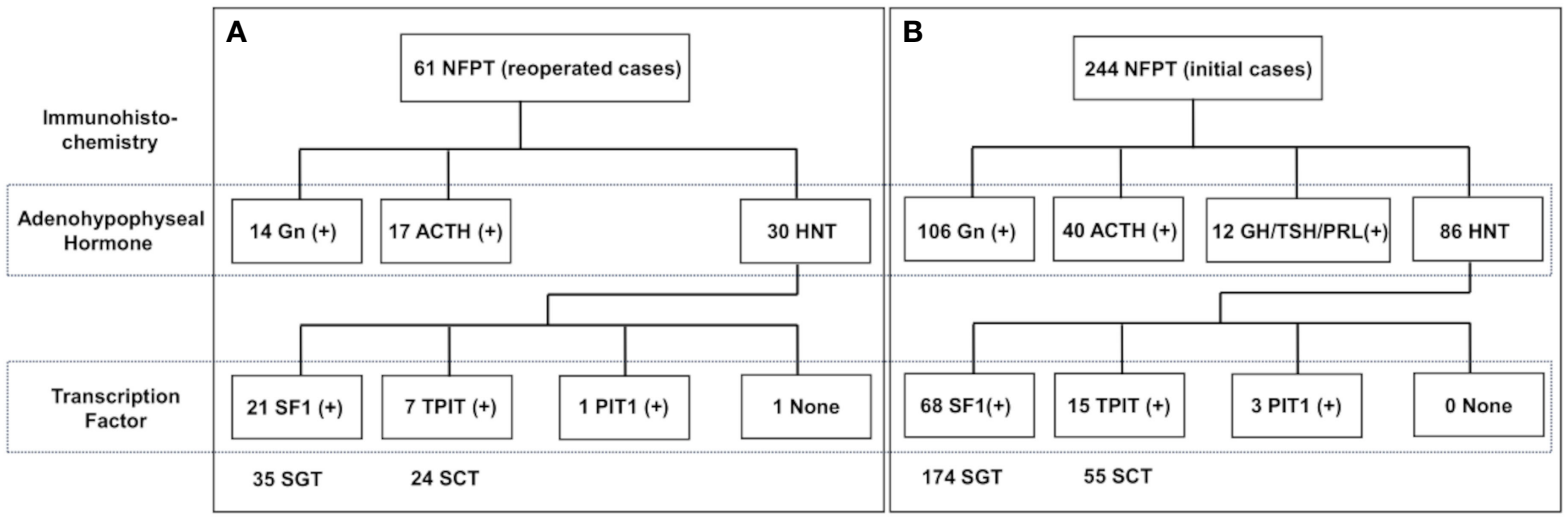
Histological classification of NFPT divided into 2 groups: **(A)** reoperation (n = 61) and **(B)** initial operation (n = 244). Histological classification is according to the results of immunohistochemistry. NFPT, nonfunctioning pituitary tumor; Gn, gonadotropin; ACTH, adrenocorticotrophic hormone; GH, growth hormone; TSH, thyrotropin stimulating hormone; PRL, prolactin; HNT, hormone-negative tumor; SF1, steroidogenic factor 1; TPIT, T-box transcription factor; PIT1, pituitary transcription factor 1; SGT, silent gonadotroph tumor; SCT, silent corticotroph tumor.

**Figure 1** demonstrates the histological classification of the reoperation (**Figure 1A**) and initial (**Figure 1B**) groups based on IHC results. There were 35 silent gonadotroph tumors (SGTs) (H-/TF+: 21), 24 silent corticotroph tumors (SCTs) (H-/TF+: 7), and 1 HNT but PIT1-positive tumor in the reoperation group. Meanwhile, there were 174 SGTs (H-/TF+: 68), 55 SCTs (H-/TF+: 15), and 3 HNT but PIT1-positive tumors in the initial operation group. The comparison of IHC results between the two groups is shown in **Table 2**. The HNT rate was significantly higher in the reoperation group. Additionally, the proportion of SCTs was significantly higher in the reoperated group than in the initial operation group. The rate of gonadotropin-negative, SF1-positive tumors among all SGTs was significantly higher in the reoperation group (**Table 2**). In the reoperation group, the mean Ki-67 LI of SCTs was slightly higher (1.35) than that of SGTs (1.09) and others (0.90), but the difference was not significant. The characteristics of the 21 patients in which the previous pathological results were available are also shown in **Table 3**. IHC was only done for adenohypophyseal hormones and not for TFs as mentioned previously. We compared the immunoreactivity between the current and the previous specimen to prove the validity of this study. There were only three patients who had discordant results. IHC for adenohypophyseal hormones was negative previously but positive this time in those three cases (**Table 3**). As a result, 12 patients were diagnosed as NCT previously (**Table 3**).

**Table 2:** Statistical Analysis of immunohistochemical results.

	Reoperated cases			Initial cases			Difference between the two groups	P value *
	Total N	n	ratio	Total N	n	ratio	% [95%CI]	
HNT/Total	61	30	49.2 %	244	86	35.3 %	13.9% [0.1-27.8%]	0.0450
SCT/Total	61	24	39.3 %	244	55	22.5 %	16.8% [3.5-30.1%]	0.0074
Gn-/Total SF1+	35	21	60.0 %	174	68	39.1 %	20.9% [3.1-38.7%]	0.0224

HNT, hormone-negative tumor; SCT, Silent corticotroph tumor; Gn, gonadotropin; SF1, steroidogenic factor 1; CI, confidence interval. * Pearson’s chi-squared test was conducted to compare proportions between the two groups.

**Table 3:** Immunohistochemistry (IHC) of all patients with available previous pathological data.

Case	EOR of previous operation	IHC of reoperation	IHC of previous operation/ previous pathological diagnosis	Comparison of those two IHC results
1	GTR	ACTH+	ACTH+/ SCT	concordant
2	GTR	ACTH+	ACTH+/ SCT	concordant
3	GTR	ACTH+	ACTH+/ SCT	concordant
4	GTR	ACTH+	None/ NCT	discordant
5	GTR	HN/ TPIT+	None/ NCT	concordant
6	GTR	FSH+	None/ NCT	discordant
7	GTR	HN/ SF1+	None/ NCT	concordant
8	GTR	HN/ SF1+	None/ NCT	concordant
9	GTR	HN/ SF1+	None/ NCT	concordant
10	GTR	HN/ SF1+	None/ NCT	concordant
11	GTR	HN/ SF1+	None/ NCT	concordant
12	GTR	None	None/ NCT	concordant
13	STR	ACTH+	ACTH+/ SCT	concordant
14	STR	ACTH+	ACTH+/ SCT	concordant
15	STR	ACTH+	ACTH+/ SCT	concordant
16	STR	ACTH+	None/ NCT	discordant
17	STR	HN/ PIT1+	None/ NCT	concordant
18	STR	HN/ SF1+	None/ NCT	concordant
19	PR	FSH+	FSH+/ SGT	concordant
20	PR	FSH+	FSH+/ SGT	concordant
21	PR	FSH+	FSH+/ SGT	concordant

IHC was only done for adenohypophyseal hormones and not for TFs in all previous pathological studies. EOR, extent of resection; GTR, gross total resection; STR, subtotal resection; PR, partial resection; ACTH, adrenocorticotrophic hormone; HN, hormone negative; TPIT, T-box transcription factor; FSH, follicle-stimulating hormone; SF1, steroidogenic factor 1; TFs, transcription factors; SCT, silent corticotroph tumor; NCT, null cell tumor; SGT, silent gonadotroph tumor.

### Ki-67 LI and duration of symptomatic regrowth by subgroup

**Figure 2:**
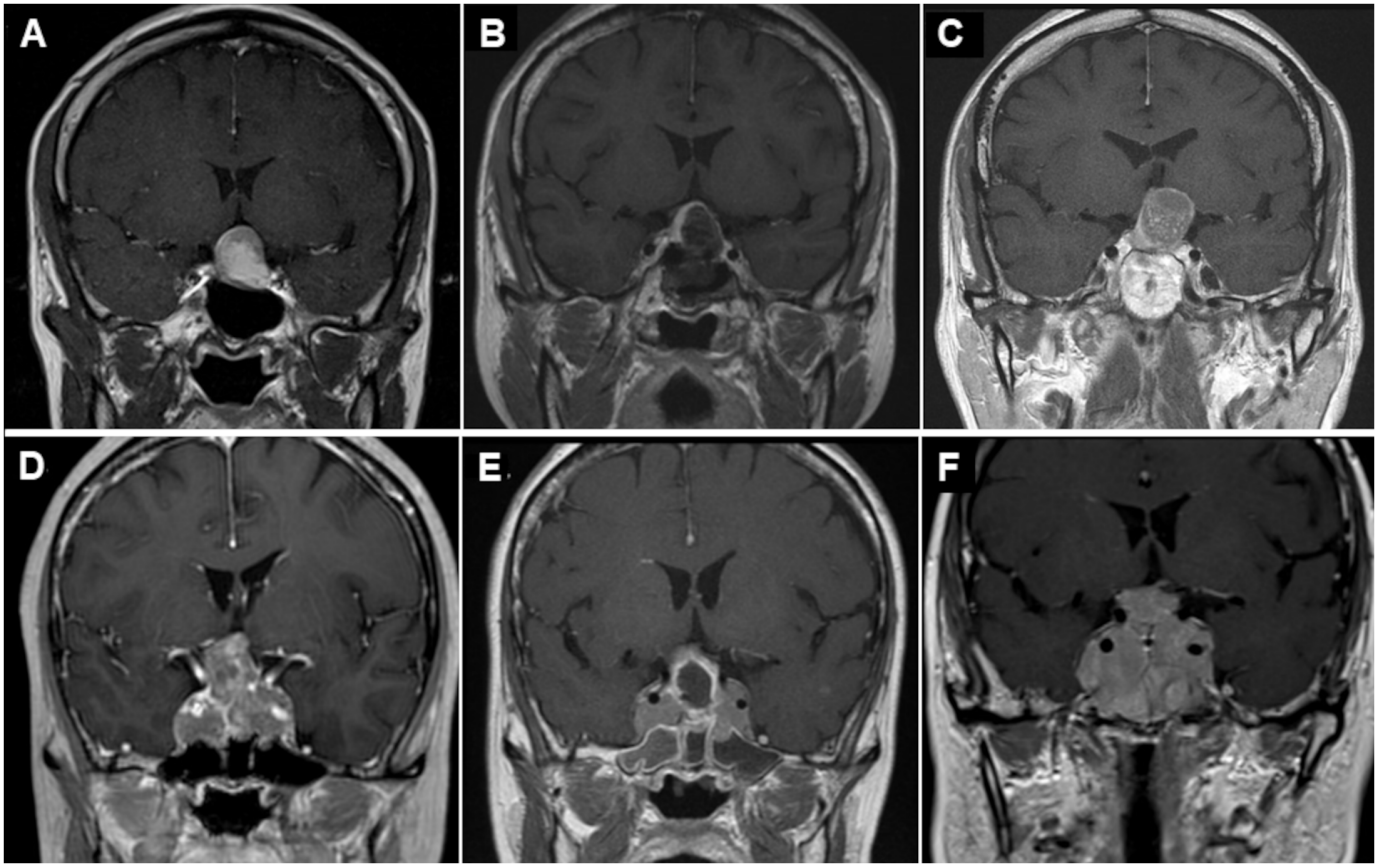
Representative case of regrowing nonfunctioning pituitary tumors after prior TSS. GTR case **(A–C)** and PR case **(D–F)**. Preoperative **(A)** and postoperative **(B)** enhanced T1-weighted MR images showing GTR after initial surgery. However, enhanced T1-weighted MR image before reoperation **(C)** showing tumor regrowth and compression of the optic nerve again 137 months after the primary surgery. Preoperative **(D)** and postoperative **(E)** enhanced T1-weighted MR images showing PR after initial TSS. Preoperative enhanced T1-weighted MR image before reoperation **(F)** showing tumor regrowth and compression of the optic nerve again 80 months after the primary surgery. TSS, transsphenoidal surgery; GTR, gross total resection; PR, partial resection; MR, magnetic resonance.

The demographics of the subgroups classified by EOR of the previous TSS are shown in **Table 4**. Some patients with STR and PR had already undergone two or more surgeries and/or radiotherapy (RT) at the previous hospitals. The Ki-67 LI of the 7 patients who had previously undergone RT in the reoperation group was 1.27, and it was not elevated after RT. Two patients in the reoperation group had Ki-67 LI > 3%, but the majority had Ki-67 LI < 2%. Ki-67 LI was higher in the GTR subgroup than in the PR and STR subgroups, although the difference was only significant for the PR subgroup (**Table 4**). Ki-67 LI values were also higher in the STR subgroup than in the PR group, although the difference was not significant. The period from the previous operation to our repeat operation was longer in the GTR subgroup than in the PR subgroup, although the difference was not significant (**Table 4**). Representative comparisons of regrown NFPTs between GTR and PR patients are shown in **Figure 2**. Enhanced T1-weighted MR images the GTR case (**Figures 2A–C**) and PR case (**Figures 2D–F**), respectively, illustrate tumor regrowth and compression of the optic nerve. The GTR case with Ki-67 LI: 2.2 regrew symptomatically in 137 months, while the PR case with Ki-67 LI: 0.4 showed symptomatic regrowth in 80 months.

**Table 4:** Statistical Analysis among different extent of the previous operation.

EOR of the previous TSS (total: 61)	GTR (n=12)	STR (n=17)	PR (n=32)	*p-value
Mean age (y) (range)	57.1 (21-80)	57.8 (39-75)	59.8 (34-77)	
Gender, % of female	41.7	58.8	40.6	
Mean value of Ki-67 LI (range)	1.81 (0.5-3.2)	1.34 (0.3-6.7)	0.87 (0.1-2.7)	GTR vs STR: 0.4272 GTR vs PR: 0.0171 STR vs PR: 0.2447
Mean number of the previous operations (times)	1	1.3	1.6	
RT after the previous operation	1	4	2	
Time to reoperation from the previous operation (mean of months) (range)	141 (35-219)	121 (28-240)	94 (8-260)	GTR vs STR: 0.7421 GTR vs PR: 0.1798 STR vs PR: 0.5185

Ki-67 LI, Ki-67 labeling index; GTR, Gross total resection; STR, Subtotal resection; PR, Partial resection. * P-values for the comparison of the two groups were calculated by Tukey's test, which accounts for multiplicity. Under line indicates statistical significance.

## Discussion

### Current problem and significance of this study

This study found the characteristics of NFPT with symptomatic regrowth and provides further insight into how these cases should be followed. NFPT regrowth is a medical challenge in the management of pituitary tumors. The number of recurrent or regrowing tumors must be analyzed to predict the type of NFPT regrowth postoperatively. In the 4th edition of the WHO classification, immunostaining for TFs was newly added to achieve a more confident pathological diagnosis of NFPT; tumors that were negative for both conventional adenohypophyseal hormones and TFs were only diagnosed as NCT (7–9). We thoroughly reviewed previous literature that investigated the relationship between histopathological classification and recurrence or regrowth in NFPT, but the results were inconsistent and debatable. These data are summarized in **Table 5** (10–16, 24, 25). Notably, there were many studies in which the NCT accounted for more than 10% of the entire NFPT. The possible causes of this inconsistency include IHC without TFs and the small number of surgical cases of regrowth. In addition, the long-term recurrence rate after GTR is low (6, 11). The reoperation rate is lower in high-volume centers with experienced surgeons, and the data on pathological results are limited to be statistically significant (11). Thus, academic discussion is difficult. At our institution, we encounter many cases of regrown tumors requiring reoperation. In the past 5 years, we have obtained solid pathological results, in all 61 cases of reoperation including immunostaining for TFs. We compared the characteristics of the reoperated cases with those of the initial surgical cases (n = 244). We also compared the immunoreactivity between the current and the previous specimen to prove the validity of this study. There were only three patients who had discordant results, in which IHC for adenohypophyseal hormones was negative previously but positive this time. This may be due to better antibodies and technical improvement from the previous pathological study which was conducted a long time ago. Thus, our strategy to use the current pathological results should be optimal.

**Table 5:** Previous literature on the relationship between histopathological classification and recurrence or regrowth in nonfunctioning pituitary tumor (NFPT).

Authors & Year	No. of Patients/ Tumor subtype	% Patients with GTR	Used antibodies for TF	No. of Patients with F/U	F/U period (months)	No. of Patients with Recurrence or Regrowth	Statistical significance of higher risk for recurrence or regrowth
Taguchi et al., 2023 (10)	191/SGT, 61/NCT, 22/SCT, 18/PIT1 lineage	SGT: 89.5%, NCT: 80.3%, SCT: 81.8%, PIT1 lineage: 83.3%	PIT1, TPIT, GATA3	113	92	SGT: 3, SCT: 1, NCT: 4, PIT1 lineage: 1	None
McClure et al., 2023 (11)	148/NFPT	All GTR	PIT1, SF1, TPIT	148	142	SGT: 7, SCT: 5, NCT: 0	None
Chatrath et al., 2022 (12)	141/SGT, 48/SCT, 23/NCT	SGT: 52.5%, SCT: 66.0%, NCT: 50.0%	Pit1, SF-1, TPIT	117	106	SGT was more likely to progress following STR compared to NCT.	None
Haddad et al., 2020 (13)	SGT/149, NCT/107	SGT: 59.7%, NCT: 53.3%	SF1. PIT1, TPIT for some cases	146	15	NCT:10 (9.3%), SGT: 4 (2.7%)	NCT > SGT
Batista et al., 2018 (14)	112/SGT, 29/SCT, 239/NCT	Not stated	None	410	79.3	SGT:36 (32%), NCT: 74 (31%), SCT: 16 (55%), PIT1 lineage: 11 (37%)	SCT > others
Almeida et al., 2019 (15)	38/SGT, 31/NCT	NCT: 61% SGT: 87%	PIT1, SF1, TPIT (since 2017)	63	60	5-year progression-free survival, NCT: 0.70 vs. SGT:1.00 (p=0.011)	NCT > SGT
Jiang et al., 2021 (16)	198/SGT, 112/SCT	SGT: 66.2%, SCT: 66.1%	PIT1, SF1, TPIT	310	13.7	Recurrence SCT: 1 (0.9%), SGT: 0 Regrowth SCT: 10 (9%), SGT: 13 (6.6%)	None
Strickland et al., 2021 (24)	100/SCT, 841/others	SCT: 42%, others: 48.3%	None	941	44.1	Recurrence SCT: 0, others: 18 (2.1%) Regrowth SCT: 12 (12%), others: 83 (9.9%)	Mean time to disease progression was significantly shorter in SCT than in others.
Carbonara et al., 2023 (25)	41/Gn+ & SF1+, 13/Gn- & SF1+	Not stated	SF1	54	Not stated	Gn+ & SF1+: 4 (9.8%) Gn- & SF1+: 3 (23.1%)	None

Gn= gonadotropin, TPIT=T-box transcription factor, PIT1=pituitary transcription factor 1, SGT=silent gonadotroph tumor, SCT=silent corticotroph tumor, GTR = gross total resection, NCT=null cell tumor, TF=transcription factor, SF1= steroidogenic factor 1, F/U=follow up.

### Diagnosis of NCT and regrowth

In our recent study, no “true NCT” was detected among 1071 pituitary neuroendocrine tumors (PitNETs), and we emphasized the importance of precise diagnosis following the most recent criteria to improve therapeutic success (21, 26). There are several NCT cases in other reports (**Table 5**). IHC is an ambiguous technique, and the results are susceptible to specimen degeneration due to events such as extended time spent at room temperature or damage via surgical manipulation. In our hospital, according to Tayler and Shi (27), tumor samples are immediately placed in 10% neutral-buffered formalin instead of immersing it in physiological saline. Similarly, Ki-67 LI must be measured carefully. To eliminate vascular endothelial cells and lymphocytes that exhibit false positives, Ki-67 LI is calculated by visual measurement under a high-magnification microscope rather than calculated using an automatic analyzer. Epigenomic analysis also suggests that the incidence of TF–negative and hormone-negative PitNETs has markedly decreased owing to advanced diagnostic techniques (28).

### Regrowth after GTR

The Ki-67 LI of GTR patients in the current study was lower than previously reported, including the proportion of patients with LI ≥ 3 (6). Tumors with relatively high Ki-67 LI and those with LI < 3 have a higher risk of regrowth in the long term, as observed in GTR cases. Interestingly, our 12 GTR patients had similar immunohistochemical characteristics to those of the 12 patients with recurrence after GTR reported by McClure et al., with 7 and 5 of them having SGT and SCT, respectively. Meanwhile, 21 of the 35 SGTs (60%) in our study were diagnosed as H-/TF (SF1) + tumors. The case without immunoreactivity to any pituitary hormones in NFPT may indicate a higher risk of regrowth after STR and PR and even after GTR. This may be one of the reasons for the higher rate of recurrence in NCT when an IHC analysis of TFs is not available. Similarly in our study, the previous pathological results diagnosed 12/21 patients as NCT, which mostly turned out to be H-/TF+ tumors (**Table 3**).

### Hormone-negative SGT and regrowth

Silva-Ortega et al. reported that using SF1 IHC enabled the detection of a substantial portion of gonadotroph tumors and reduced the estimated prevalence of NCTs to less than 5% (29). Thus, the many NCTs reported to have more recurrence and regrowth could be reclassified as hormone-negative, SF1-positive SGTs. Carbonara et al. recently reported that hormone-negative SGTs were not significantly different from other SGTs concerning invasion or proliferation patterns (25). The recurrence rate was higher in hormone-negative SGTs than in other SGTs, although the difference was not significant due to their small number of cases (25). In the current study, the rate of H-/TF+ tumors among SGTs was significantly higher in the reoperation group than in the initial operation group. The reason for this is unknown, but H-/TF+ tumors may be less differentiated than anterior pituitary hormone-positive tumors, as reported for PIT1 lineage tumors (30).

### SCT and regrowth

SCTs are considered a “high-risk” subtype of pituitary tumors in the 2017 WHO classification system (24, 31, 32). However, this is controversial, and conflicting findings have been reported (33–35). For example, Chatrath et al. recently reported that compared to both corticotroph and null cell adenomas classified using the 2017 WHO guidelines, gonadotroph adenomas were likely to progress following STR (12). However, these differences did not reach significance (12). Again, the inconsistency may be due to the lack of accuracy in IHC and/or the small number of samples. Strickland et al. defined SCT only as ACTH immunoreactivity and did not include TPIT IHC (24). There were several ACTH-negative but TPIT-positive SCTs in our study (7/24 SCT). Thus, ACTH-negative but TPIT-positive tumors were not categorized as SCT in their report (24). To the best of our knowledge, this is the first report in which a large number of reoperation patients with reliable IHC results for both ACTH and TPIT immunostaining were compared with patients who only underwent initial surgery. Pathological examination showed significantly more SCTs in the reoperation group than in the initial operation group. Surprisingly, nearly 40% of the 61 patients with symptomatic recurrence had SCT, and this proportion was remarkably higher than that in the initial operation group (22.5%) like in other reports (10, 12, 21).

### Regrowth after PR

Surgeons should aim for GTR in NFPT (36–39), but it is neither easy nor safe (6). In the real world, countless TSS for NFPT ends up with PR (6, 14). We found that NFPT, after PR operation, regrows and becomes symptomatic again in a much shorter time, even with a significantly lower Ki-67 LI than in GTR. The reason is quite understandable: a small number of residual tumors after GTR only becomes large enough to be symptomatic with a relatively large Ki-67 LI and an extended period. In contrast, many residual tumors regrow after a PR and become symptomatic, even with a low Ki-67 LI, in a shorter time. Some studies (6, 10, 22) concluded that cavernous sinus invasion (CSI) significantly predicts postoperative progression in NFPT. CSI causes incomplete resection and increases the number of residual tumor cells. In our recent report, 57% of functioning pituitary tumors with direct contact with the medial wall of the cavernous sinus had histological CSI (40). Therefore, there may be many residual tumor cells even with GTR. Furthermore, it is technically impossible to resect all tumor cells with complete CSI (40), especially those with Knosp grade 4, resulting in PR. Thus, we classified reoperation patients according to EOR instead of CSI.

### Ki-67 LI and regrowth

The characteristics of regrowing NFPTs after operation have long been discussed. A Ki-67 LI of >3% has been reported to predict recurrence and regrowth in NFPTs (19, 20). However, in the real world, most regrowing NFPTs have a low Ki-67 LI (21, 22). Chiloiro et al. suggested that Ki-67 LI ≥ 1.5% might be useful as a prognostic marker for pituitary tumors after radical removal (41, 42). The results of this study also indicate that the reference values of Ki-67 LI regarding the risks of regrowing depend on the EOR of the previous surgeries. NFPTs with low Ki-67 LI should be carefully monitored as they may regrow to be symptomatic, especially after incomplete removal.

### Limitations

We used the results of the pathological examination of the reoperated samples. Although this should be valid, regrowing tumors from patients who underwent an initial operation in our hospital needs to be further analyzed to validate our results. However, this is time-consuming because only one patient underwent reoperation in our own cases.

## Conclusions

The ratio of H-/TF+ SGT is significantly higher in symptomatically regrown tumors than in the initial cases, which used to be diagnosed as NCT before the introduction of immunostaining for TF. With meticulous IHC of TPIT, we also confirmed that the ratio of SCT is significantly higher in cases with symptomatic tumor regrowth than in initial cases. PR cases tend to grow symptomatically in a shorter period of time, even with lower Ki-67 LI than GTR cases. On the contrary, we should follow GTR cases for a longer. especially with relatively high Ki-67 LI.

## Data availability statement

The original contributions presented in the study are included in the article/supplementary material. Further inquiries can be directed to the corresponding author.

## Ethics statement

The studies involving humans were approved by The ethical review board of Moriyama Memorial Hospital. The studies were conducted in accordance with the local legislation and institutional requirements. The participants provided their written informed consent to participate in this study. Written informed consent was obtained from the individual(s) for the publication of any potentially identifiable images or data included in this article.

## Author contributions

AI: Conceptualization, Data curation, Formal analysis, Investigation, Methodology, Project administration, Validation, Visualization, Writing – original draft, Writing – review & editing. NI: Data curation, Methodology, Supervision, Writing – review & editing. NT: Data curation, Writing – review & editing. KT: Supervision, Writing – review & editing, Conceptualization. ST-M: Data curation, Writing – review & editing, Methodology. MK: Data curation, Writing – review & editing. HY: Data curation, Writing – review & editing. HS: Data curation, Writing – review & editing. GM: Data curation, Writing – review & editing. SY: Conceptualization, Investigation, Methodology, Project administration, Supervision, Writing – review & editing.
